# Nontuberculous Mycobacterium infection complicated with Haemophagocytic syndrome: a case report and literature review

**DOI:** 10.1186/s12879-019-4061-9

**Published:** 2019-05-09

**Authors:** Wen Shi, Yang Jiao

**Affiliations:** Department of General Internal Medicine, Peking Union Medical College Hospital, Chinese Academy of Medical Sciences & Peking Union Medical College, No. 1, Shuaifuyuan, Wangfujing St, Beijing, 100730 China

**Keywords:** Nontuberculous *Mycobacterium* infection, Haemophagocytic syndrome, *M. Intracellulare*

## Abstract

**Background:**

Non-tuberculous mycobacterial (NTM) infection is usually observed in patients with immunosuppressive conditions. It may also cause unregulated immune responses. While there have been increasing numbers of reported tuberculosis-related HPS (haemophagocytic syndrome), HPS caused by NTM infection is still very rarely reported.

**Case presentation:**

We report a previously healthy 21-year-old Chinese female with fever, night sweats and fatigue, in whom HPS was diagnosed according to the HLH-2004 criteria. *Mycobacterium intracellulare* was cultured from her peripheral blood. After treatment with corticosteroid, clarithromycin, rifampicin, ethambutol and amikacin, the patient finally recovered. We also reviewed relevant publications on NTM infection complicated with HPS and found 11 cases, including ours. Clinical presentations, diagnoses and prognoses were analysed and summarized to deepen our understanding of this rare condition.

**Conclusions:**

Most reported NTM-related cases were caused by disseminated infection. The lack of localized symptoms might add to the difficulty involved in making the right diagnosis. While it usually takes time to obtain tissue or blood culture results, granuloma in a bone marrow biopsy might be an early indicator of possible mycobacterial infection. Although treatment varied, the overall prognosis of NTM-related HPS was promising.

## Background

Haemophagocytic syndrome (HPS) is a rare, life-threatening condition characterized by over-stimulation of the immune system [1]. While primary HPS is genetically predisposed and usually develops during childhood and adolescence, secondary HPS can be triggered by a variety of conditions and may occur at any age [2]. The most common causes of HPS include viral infection, autoimmune disease and haematological malignancy [3]. On the one hand, NTM infection is usually observed in patients with immunosuppressive conditions [4, 5]. On the other hand, it may cause intense immune responses. Although severe tuberculosis has been increasingly reported and recognized as a potential cause of HPS in recent years [4, 5], non-tuberculous mycobacterial (NTM) infection associated with HPS is still very rare. Here, we report a 21-year-old female who was diagnosed with HPS caused by disseminated *Mycobacterium intracellulare* infection where treatment of both NTM infection and HPS eventually led to recovery. We also performed a literature review of NTM infection complicated by HPS to deepen our understanding of the condition.

## Case presentation

A 21-year-old female was admitted to our hospital complaining of fever and fatigue for 2 months. She was an editor and had been in good health until 2 months ago when she developed a spiking fever of 39–40 °C, dry cough, night sweats and fatigue. She went to a local hospital, and a complete blood count (CBC) revealed only mild anaemia (Table 1). She was diagnosed with an “upper respiratory tract infection”. Cefprozil was administered, but her symptoms gradually worsened, and a growing mass, which was painful, was found on the right side of her neck. She reported no arthralgia, rash, weight loss or relevant family history.

**Table 1:** Laboratory results of our case

	First visit	First day after admission	1 week after admission	6-month follow-up
WBC (*10^9^/L)	4.36	2.28	1.02	3.45
NEUT (*10^9^/L)	3.66	1.77	0.71	2.87
HGB (g/L)	91	87	59	112
PLT (*10^9^/L)	109	71	51	141
ALT (U/L)		192		7
AST (U/L)		139		11
LDH (U/L)		554		
Cr (μmol/L)		89		69
ESR (mm/h)		> 140		
hsCRP (mg/L)		174		7.9
Triglyceride (mmol/L)		2.62		
Fibrinogen (g/L)		3.5		
Fer (ng/ml)		4090		
B cell (/μl)		7		56
T cell (/μl)		233		459
NK cell (/μl)		6		89
Other	CMV-DNA, EBV-DNA, CMV-IgM, CMV-pp65, HIV antibody, T.SPOT-TB(−) ANA, ANCA, anti-RNP, anti-Sm, anti-SSA, anti-SSB, anti-rRNP, anti-Scl-70, anti-Jo-1, anti-PM-Scl, anti-dsDNA, anti-PCNA, ACA, AMA-M2, anti-histone antibodies, anti-CCP, ACL, anti-β2GP1, LAC, Coombs(−)			

WBC, white blood cells; NEUT, neutrophils; HGB, haemoglobin; PLT, platelets; ALT, alanine aminotransferase; AST, aspartate aminotransferase; LDH, lactate dehydrogenase; Cr, creatinine; ESR, erythrocyte sedimentation rate; hsCRP, high-sensitivity C-reactive protein; Fer, ferritin; SF, serum ferritin; ANA, antinuclear antibody; ANCA, antineutrophil cytoplasmic antibodies; T.SPOT-TB, interferon-gamma release assay T.SPOT-TB; anti-RNP, anti-ribonucleoprotein antibodies; anti-Sm, anti-Smith antibody; anti-SSA, anti-Sjögren syndrome A antibody; anti-SSB, anti-Sjögren syndrome B antibody; anti-rRNP, anti-ribosomal RNP antibodies; anti-Scl-70, anti-Scl-70 antibody; anti-dsDNA, anti-double-stranded-DNA antibody; anti-Jo-1, anti-Jo-1 antibody; anti-PM-Scl, anti-exosome antibodies; anti-PCNA, anti-proliferating-cell-nuclear-antigen antibody; ACA, anticentromere antibody; AMA-M2, antimitochondrial antibody M2 subtype; CCP, cyclic citrullinated peptide; ACL, anti-cardiolipid antibody; β2GP1, β2-glycoprotein I; LAC, lupus anti-coagulant

**Fig. 1:**
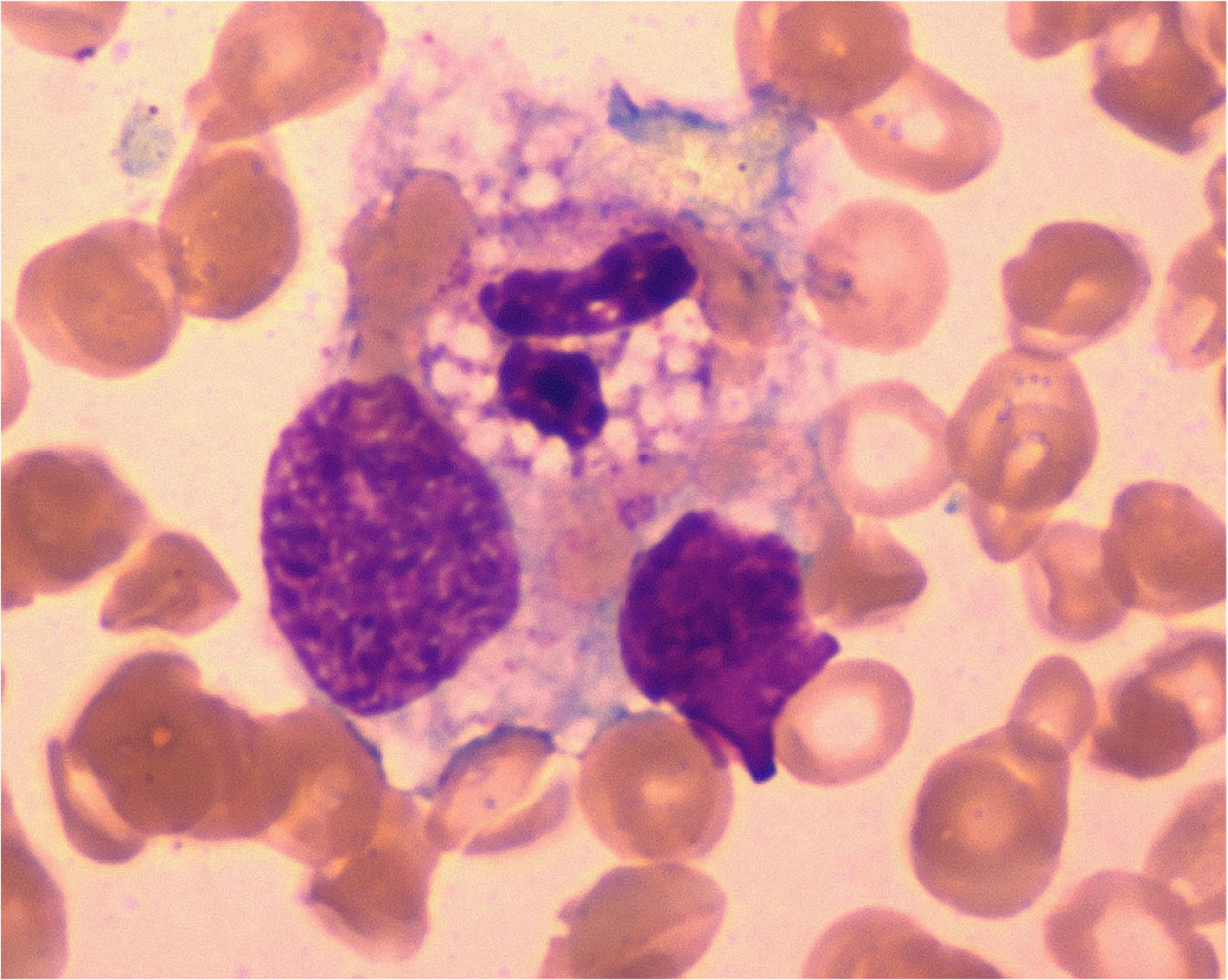
Bone marrow aspirate specimen showed a haemophagocyte engulfing a polymorphonuclear leukocyte, a lymphocyte and erythrocytes (Wright-Giemsa stain, 1000x)

**Fig. 2:**
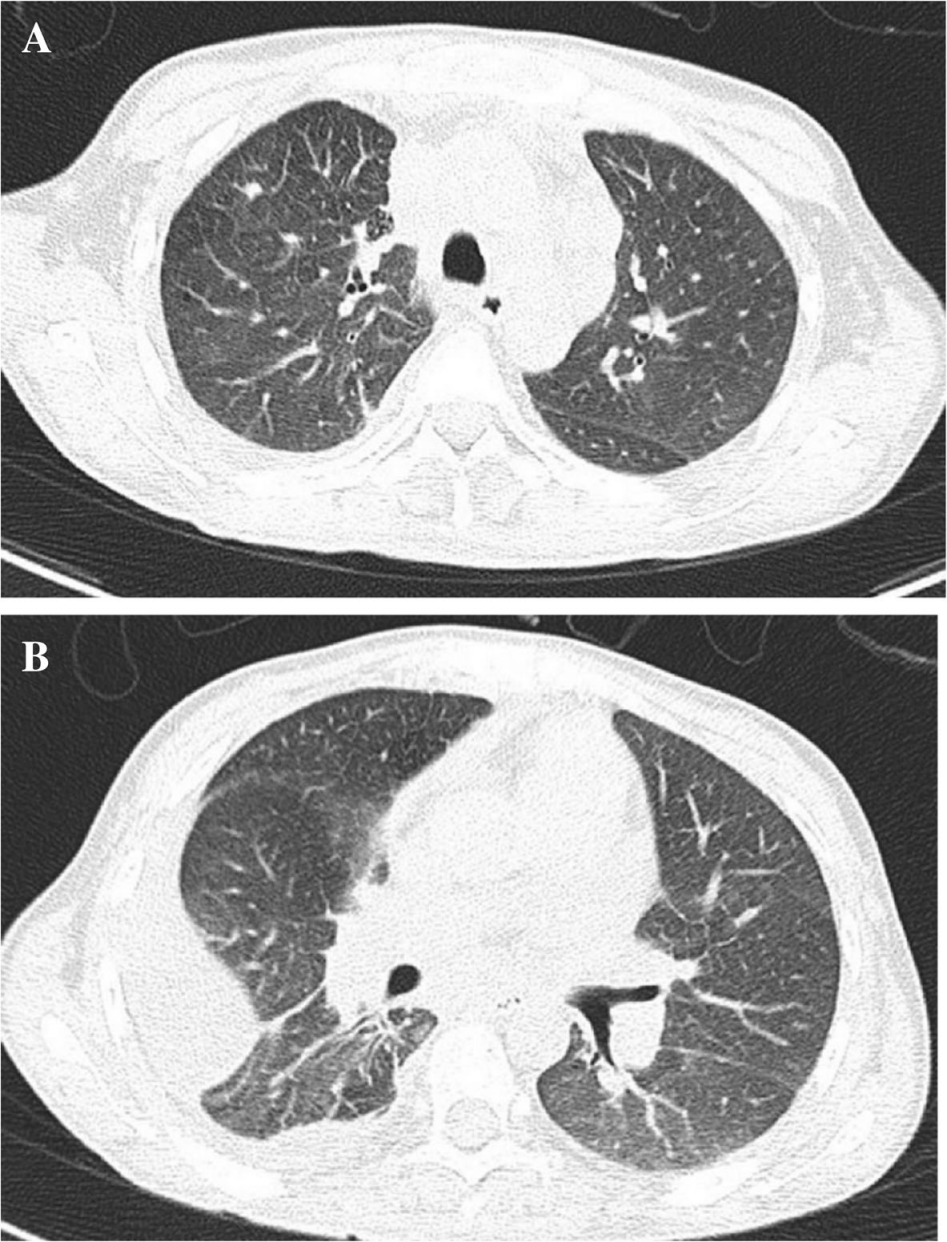
(**a**) Chest CT scan revealed scattered nodules and (**b**) right pleural effusion

Her physical examination upon admission revealed scattered rales on the right lung, splenomegaly and enlarged lymph nodes that had coalesced and were palpated in her right cervical region. The initial laboratory investigation showed peripheral pancytopenia (white blood cells (WBC) 2.28*10^9^/L, HGB 87 g/L, PLT 71*10^9^/L, Table 1), elevated liver enzymes (ALT 192 U/L, AST 139 U/L, LDH 554 U/L) and hyperferritinaemia (Fer 4090 ng/ml). Natural killer (NK) cell activity was tested via a flow cytometry-based assay previously reported by Zhang et al. and was found to be reduced [6]. Haemophagocytosis was found in the bone marrow aspirate (Fig. 1). Initial serological investigations for common pathogens and autoimmune diseases were negative (Table 1). A chest CT scan revealed bilateral nodules and right pleural effusion (Fig. 2), but Gram staining, acid-fast staining and mycobacterial culture of sputum were negative. An ultrasound-guided puncture was performed on her right cervical lymph nodes. Ziehl-Neelsen staining revealed acid-fast bacilli, and further nucleic acid probes identified *M. intracellulare*. Histology from the biopsied tissue showed inflammatory necrosis and calcification without evidence of malignancy. Then, *M. intracellulare* was cultured from her peripheral blood and lymph node tissue. We suspected that the patient might have autoantibodies against interferon-γ, which led to her immunosuppressive state and susceptibility to NTM infection. Unfortunately, serum testing for interferon-γ autoantibody was not available in our hospital.

A regimen containing clarithromycin (500 mg BID po), rifampicin (450 mg QD po), ethambutol (750 mg QD po) and amikacin (0.4 g QD iv) was started to treat the disseminated *M. intracellulare* infection. HPS was also diagnosed according to the HLH-2004 criteria, and prednisone 60 mg QD po (1 mg/kg/d) was given to control the inflammation.

Her temperature soon became normal. Prednisone was gradually tapered (60 mg QD*2 weeks→40 mg QD*2 weeks→20 mg QD*2 weeks→↓5 mg/2 weeks until stop), and amikacin was stopped after 1 month, while clarithromycin, rifampicin and ethambutol were continued. She was in remission, and her cervical lymph nodes were unpalpable with normal CBC at the 6-month follow-up visit. The patient was then lost to follow-up.

## Literature review

We then searched PubMed for cases of NTM infection complicated with HPS (see Appendix for detailed search strategy) and found 10 case reports in addition to ours (Table 2).

**Table 2:** Published cases of NTM infection complicated with HPS

	Author Country Year	Sex/Age	Symptoms	Cause of NTM infection	Method	Infection location	Underlying disease	Treatment	Prognosis	BM pathology showing granuloma
1	D. Har/USA, 2017 [12]	M/58	Fever, night sweats, wt loss	*M. kansasii*	Spleen biopsy culture	Isolated spleen lesion	Sweet’s syndrome, adalimub (2 years)	Dexamethasone, etoposide, RIPE	Death	Not mentioned
2	Chou/PRC, 2010 [13]	F/60	Fever, postprandial nausea, vomiting	*M. kansasii*	BM culture, liver biopsy culture	Disseminated	Femoral sarcoma (5 months later)	Methylprednisolone, IVIG, RIPE, azithromycin	Recovery	Yes
3	Thomas/France 2014 [14]	M/65	Fever, wt loss, diarrhoea	*M. lentiflavum*	Blood culture, faecal culture, BALF, liver autopsy	Disseminated	Heart transplantation, tacrolimus+MMF	Prednisone, CsA, MMF; rifabutin, ethambutol, clarithromycin, amikacin	Death	Yes
4	Ordaya/USA, 2017 [15]	F/26	Fever, fatigue, sore throat	*M. avium complex*	Blood culture	Disseminated	ALL in remission	Dexamethasone, etoposide, rifabutin, ethambutol, clarithromycin	Recovery (stop transplantation)	Yes
5	Hilmers/USA, 2013 [16]	M/40	Fever	*M. avium complex*	Blood culture, BALF, BM culture	Disseminated	AIDS, HAART (3 weeks, histoplasmosis)	Azithromycin, ethambutol, rifabutin, antifungal, HAART	Recovery	Not mentioned
6	Chamsi-Pasha/USA, 2013 [17]	F/22	Fever, night sweats, wt loss	*M. avium complex*	Lung biopsy culture, blood culture, BM culture	Disseminated	Sickle cell anaemia	Azithromycin, ethambutol, rifampin, methylprednisolone, anakinra	Death	Yes
7	Yang/PRC, 2003 [18]	F/15	Fever, lethargy	*M. avium complex*	Gastric lavage	Disseminated	SLE (prednisone +HCQ)	IVIG, etoposide; clarithromycin, ciproflaxcin, amikacin	Recovery	Yes
8	Lapierre/France, 2017 [19]	M/55	Fever, wt loss	*M. iranicum (multidrug-resistant)*	Blood culture	Disseminated	Hodgkin’s disease (at the same time)	Corticosteroid, BEACOPP	Recovery	Not mentioned
9	M.I/Japan, 2018 [20]	M/14	Fever, wt loss	*M. kansasii*	Blood culture, BM culture	Disseminated	MDS (3 years), GATA2 heterozygous mutation	Prednisone, etoposide, CsA, RIPE, transplant	Recovery	No
10	Katagiri/Japan, 2014 [21]	M/58	Fever, rash	*M. abscessus*	Blood culture, skin	Disseminated	CLL (prednisone +CTX)	Imipenem, amikacin, clarithromycin, levofloxacin, IVIG	Recovery	No
11	Our case	F/21	Fever, night sweats, fatigue	*M. intracellulare*	Blood culture, lymph node	Disseminated	No	Clarithromycin, rifampicin, ethambutol, amikacin, prednisone	Recovery	No

BM: Bone marrow; wt: weight; R: rifampin; I: isoniazid; P: pyramide, E: ethambutol; IVIG: intravenous immunoglobin; BALF: bronchoalveolar lavage fluid; MMF: mycophenolate; CsA: cyclosporin A; HAART: highly active antiretroviral therapy; BEACOPP: bleomycin, etoposide, adriamycin, cyclophosphamide, oncovorin, procarbazine and prednisone; MDS: myelodysplastic syndromes; GATA2: GATA-binding factor 2 gene; CLL: chronic lymphocytic leukaemia

All patients except ours were found to have concomitant immunosuppressive conditions: 1 with AIDS, 2 with autoimmune diseases (1 systemic lupus (SLE) and 1 Sweet’s syndrome, both taking immunosuppressive agents), 1 who had undergone an organ transplantation, 1 with sickle cell anaemia and 4 with haematological malignancies (1 acute lymphoblastic leukaemia (ALL), 1 chronic lymphocytic leukaemia (CLL), 1 myelodysplastic syndrome (MDS) + *GATA2* gene mutation and 1 Hodgkin’s lymphoma). One patient was found to have advanced femoral sarcoma 5 months after NTM infection, and HPS was diagnosed.

Most patients (9/11) definitely had disseminated NTM infections. In case 7, however, although the *M. avium complex* was cultured only from gastric lavage and blood culture was negative, non-caseating granulomatosis was found in a bone marrow biopsy, indicating that this might be a disseminated *M. avium complex* infection. Similar to our patient, all patients reported constitutional symptoms (fever, night sweats, weight loss, fatigue, etc.) as their main complaints. Other symptoms were rather vague and nonspecific except in case 10, where the patient was found to have rashes, and skin biopsy culture revealed *M. abscessus*.

For pathogenic NTM infection, *M. avium complex* (including *M. intracellulare* in our patient) was found in 5 patients, *M. kansasii* in 4 patients, *M. lentiflavum* in 1 patient, *M. iranicum* in 1 patient and *M. abscessus* in 1 patient.

Like our patient, most patients (9/11) were first suspected of having and diagnosed with HPS before NTM infection was revealed in blood or tissue specimens. The exceptions included case 3, whose faecal specimen had positive *M. lentiflavum* PCR results but was left untreated until HPS developed when the patient was re-evaluated, and case 6, who had been treated for *M. avium* complex infection but later deteriorated with the development of HPS. Interestingly, 5/11 patients were found to have granuloma in the bone marrow specimen before NTM infection finally developed from concomitant blood or tissue specimen culture.

Treatment varied considerably. Corticosteroid, etoposide, cyclosporin A (CsA) and intravenous immunoglobulin (IVIG) were administered in different combinations to treat HPS in most patients. Case 6 was also treated with anakinra in addition to corticosteroids. Antibiotics were added to treat NTM infection and adjusted according to speciation. There were, however, 2 exceptions. Case 5 had been diagnosed with acquired immune deficiency syndrome (AIDS) and started highly active antiretroviral therapy (HAART) 3 weeks before disseminated *M. avium complex* and histoplasmosis complicated with HPS were diagnosed. Immunosuppressive therapy was not added for HPS, but antimycobacterial and anti-fungal drugs were used as well as HAART. The patient eventually recovered. Case 8 was diagnosed as disseminated *M. iranicum* infection complicated by HPS. Lymph node biopsy revealed a stage IV Hodgkin’s disease at the same time. Since his *M. iranicum* was multidrug-resistant, he was treated only with bleomycin, etoposide, adriamycin, cyclophosphamide, oncovorin, procarbazine and prednisone (BEACOPP) for his HPS and Hodgkin disease. The patient also recovered. The prognoses of patients with reported NTM infection complicated with HPS were relatively good: 2/11 died, while 9/11 survived. In case 4, the correct diagnosis of NTM infection as the cause of HPS instead of ALL relapse even prevented haematopoietic stem cell transplantation.

## Discussion and conclusions

NTM infection is caused by generally free-living organisms that are ubiquitous in the environment [7]. With the development of new microbiological methods, NTM infection in human diseases has become increasingly recognized and reported [8]. *M. avium complex* and *M. kansasii* are believed to be the most common species [9], which is in accordance with our literature review results. On the one hand, NTM infection, especially disseminated NTM infection, is usually observed in patients with immunosuppressive conditions [9]; on the other hand, NTM infection can trigger HPS, a strong and uncontrolled immune response. Our literature review found 11 cases (including ours) in total indicating that the correlation is still rarely reported, and all cases were reported after 2010. However, secondary HPS is often associated with intracellular bacteria that induce classical Th1 immune responses, which, in animal models, are needed for the control of tuberculosis infection [10]. This might also be the reason why NTM infection can cause HPS.

Most of the reported patients, including ours, had disseminated NTM infection. All patients except ours had known underlying immune-compromised conditions. Our initial evaluation of the patient’s immune state showed that the counts of B cells, T cells and NK cells were all decreased, while serum immunoglobulin levels were within normal ranges. After treatment, the patient’s B cell, T cell and NK cell counts gradually rose. Thus, we doubt the decreases in the patient’s lymphocytes and NK cells might be the result, rather than the cause, of her NTM infection. However, we cannot rule out the possibility that our patient might have undiscovered immunosuppressive conditions. Interestingly, case 2 was found to have advanced femoral sarcoma 5 months after being diagnosed with NTM infection and HPS. However, our patient was lost to follow-up 6 months later, and a more thorough investigation of possible underlying diseases, for example, malignancy, was not performed. As mentioned before, serum testing for interferon-γ autoantibody was not available in our hospital until recently. Thus, we were not able to verify our suspicions before the patient was discharged and then lost to follow-up. Therefore, it is recommended that patients who are diagnosed with NTM infection complicated by HPS are carefully investigated to determine possible concomitant immune-compromised conditions.

*M. intracellulare* was grown from both the blood and cervical lymph node biopsy tissue cultures of our patient. Her chest CT scan also revealed bilateral nodules and right pleural effusion, indicating possible pulmonary involvement, even though no pathogens were successfully grown from the patient’s sputum. No other focus of NTM involvement was found. *M. intracellulare* is reported to exist naturally in the environment, and the lung is the most common organ involved in *M. intracellulare* infection. Thus, we suspect that the port of entry of the mycobacteraemia might be from the respiratory tract, as is usually seen in patients with disseminated *M. intracellulare* infection. All patients reported constitutional symptoms as major complaints, which was in accordance with the fact that most cases were disseminated and lacked specific localized symptoms. In the majority of patients, HPS was suspected and diagnosed before NTM infection was detected and speciated. Interestingly, nearly half of the patients were found to have granuloma from bone marrow biopsy, which Is usually a routine examination when HPS is suspected. This biopsy finding could often be revealed earlier than the culture result, making it a promising positive predictor of disseminated mycobacterial (TB [11] or NTM) infection as the cause of HPS. In immunocompromised patients, HPS plus granuloma from bone marrow biopsy might justify a more thorough investigation and suspicion of possible mycobacterial infection.

Although treatment varied considerably, especially for HPS, the overall prognoses of NTM infection complicated with HPS seemed promising. Eight out of 11 patients recovered after treatment, and the correct diagnosis of NTM infection as the cause of HPS instead of ALL relapse even prevented haematopoietic stem cell transplantation in 1 patient, further indicating the importance of timely diagnosis of NTM infection complicated with HPS and starting treatment as soon as possible.

In conclusion, NTM infection is a rare cause of secondary HPS. Here, we report a 21-year-old female with disseminated *M. intracellulare* infection complicated by HPS who was successfully treated with antibiotics and corticosteroid. We reviewed relevant publications of NTM infection with HPS. Most reported NTM-related cases were caused by disseminated infection. Lack of localized symptoms might add to the difficulty involved in making the right diagnosis. While it usually takes time to obtain tissue or blood culture results, granuloma in a bone marrow biopsy might be an early indicator of possible mycobacterial infection. Although treatments varied, when patients were treated in a timely manner with antibiotics and anti-inflammation therapy, the overall prognosis of NTM-related HPS was promising.

## Abbreviations

ACA: Anticentromere antibody
ACL: Anti-cardiolipid antibody
AIDS: Acquired immune deficiency syndrome
ALL: Acute lymphoblastic leukaemia
ALT: Alanine aminotransferase
AMA-M2: Antimitochondrial antibody M2 subtype
ANA: Antinuclear antibody
ANCA: Antineutrophil cytoplasmic antibodies
Anti-dsDNA: Anti-double-stranded-DNA antibody
Anti-Jo-1: Anti-Jo-1 antibody
Anti-PCNA: Anti-proliferating-cell-nuclear-antigen antibody
Anti-PM-Scl: Anti-exosome antibodies
Anti-RNP: Anti-ribonucleoprotein antibodies
Anti-rRNP: Anti-ribosomal RNP antibodies
Anti-Scl-70: Anti-Scl-70 antibody
Anti-Sm: Anti-Smith antibody
Anti-SSA: Anti-Sjögren syndrome A antibody
Anti-SSB: Anti-Sjögren syndrome B antibody
AST: Aspartate aminotransferase
BALF: Bronchoalveolar lavage fluid
BEACOPP: Bleomycin, etoposide, adriamycin, cyclophosphamide, oncovorin, procarbazine and prednisone
BM: Bone marrow
CBC: Complete blood count
CCP: Cyclic citrullinated peptide
CLL: Chronic lymphocytic leukaemia
Cr: Creatinine
CsA: Cyclosporin A
E: Ethambutol
ESR: Erythrocyte sedimentation rate
Fer: Ferritin
*GATA2*: *GATA-binding factor 2* gene
HAART: Highly active antiretroviral therapy
HGB: Haemoglobin
HLH: Haemophagocytic lymphohistiocytosis
HPS: Haemophagocytic syndrome
hsCRP: High-sensitivity C-reactive protein
I: Isoniazid
iv: Intravenous
IVIG: Intravenous immunoglobulin
LAC: Lupus anti-coagulant
LDH: Lactate dehydrogenase
*M. abscessus*: *Mycobacterium abscessus*
*M. avium complex*: *Mycobacterium avium complex*
*M. intracellulare*: *Mycobacterium intracellulare*
*M. iranicum*: *Mycobacterium iranicum*
*M. kansasii*: *Mycobacterium kansasii*
*M. lentiflavum*: *Mycobacterium lentiflavum*
MDS: Myelodysplastic syndrome
MMF: Mycophenolate
NEUT: Neutrophils
NK: Natural killer
NTM: Non-tuberculous mycobacterial
P: Pyramide
PCR: Polymerase chain reaction
PLT: Platelets
R: Rifampin
SF: Serum ferritin
SLE: Systemic lupus
T.SPOT-TB: Interferon-gamma release assay T.SPOT-TB
WBC: White blood cells
wt: Weight
β2GP1: β2-glycoprotein I
